# Impact of Comprehensive Geriatric Assessments on Dementia Care

**DOI:** 10.3390/geriatrics11020039

**Published:** 2026-04-01

**Authors:** Shazia Durrani, Minhal Mussawar, Mariam Alaverdashvili

**Affiliations:** 1 Department of Psychiatry, College of Medicine, University of Saskatchewan, Saskatoon, SK S7K 0M7, Canada; mariam.alaverdashvili@usask.ca; 2 Saskatchewan Health Authority, Saskatoon, SK S7K 0M7, Canada; 3 College of Medicine, University of Saskatchewan, Saskatoon, SK S7K 0M7, Canada

**Keywords:** comprehensive geriatric assessment, dementia, elderly

## Abstract

**Introduction:** According to the Alzheimer Society of Canada, over 770,000 people in Canada are living with dementia. This number is expected to rise to nearly 1 million people by 2030. Although the provision of team-based interprofessional assessment in gerontological care is critical for the early detection and prevention of dementia, its planning and delivery can be a challenge. In Saskatchewan, previous assessments have identified significant gaps between actual and best practices in dealing with this medical condition. The emergence of Geriatric Services Resource Teams (GSRTs), which apply an innovative, team-based model to improve the diagnosis and care of older adults with complex health practices, can be proven beneficial in this regard. The purpose of this study is to compare the efficacy of the care provision process between a GSRT and a traditional medical care channel (i.e., primary health) with respect to dementia patients. **Methods:** A retrospective patient chart review was conducted by collecting data from a large Primary Care practice (*n* = 90) and the GSRT in Regina (*n* = 75). Collected data included information on patient demographics and treatment, and the diagnosis process itself. **Results:** While demographic characteristics between patient groups were similar, significant differences (*p* < 0.05) were found in the involvement of pharmacy and other healthcare professionals, prescriptions for memory loss, and in who made the diagnosis. Moreover, although the dementia diagnosis was usually made first in Primary Care, further clarification of the type of dementia, counseling of diagnosis, review of medication, and assessment of functions and social supports were better managed in the GSRT group. **Discussion:** The use of Geriatric Services Resource Teams is a relatively new concept in Saskatchewan. As these teams are established, initial results show that their role in complex care management has beneficial outcomes for dementia patients.

## 1. Introduction

Canadians aged 65 and above make up less than one-fifth of the population, yet utilize nearly half of all healthcare spending [1,2,3]. Reasons for this include a healthcare system focused on managing acute rather than chronic diseases (which are seen more frequently in the elderly), inadequate support in long-term care settings, a higher cost of living and the increased risk for multiple comorbidities as people age [4]. Other factors include the increasing prevalence of Mild Cognitive Impairment (MCI) and dementia in the elderly population [5], with these patients presenting with additional care needs compared to those without. The variability in the clinical presentation, detection, and symptoms associated with both dementia and prodromal MCI [4] make it even more challenging to ensure appropriate diagnosis and management. In Canada, additional factors including the geography of low population density, combined with climate challenges like severe winters, create barriers to healthcare access, contributing to disparities in physician density and specialist availability between urban and rural areas [6]. This can lead to insufficient staffing, poor job quality and insufficient skills, which have a toll on the quality of care and safety [7]. To provide care to elderly people, various care models have been created and implemented to improve health, care utilization and cost moderation, highlighting the necessity of innovative models to improve healthcare outcomes for older adults by focusing on comprehensive geriatric assessment, evidence-based interventions, and care coordination [8]. The Comprehensive Geriatric Assessment (CGA) has become the cornerstone of clinical care for elderly because it provides inter-disciplinary assessments for patients with complex needs [9,10]. In Canada, the family physician is typically the first point of contact in the healthcare system for most patients; however, studies report that family physicians feel ill-equipped in providing thorough dementia care [11,12]. Some of the reasons for this include navigating a complicated relationship between the biomedical, psychosocial, and ethical aspects of the condition, perceived knowledge and skill gaps [10], financial constraints, and a lack of awareness of dementia support services [13].

Regina is an urban center in Saskatchewan, Canada, where the Geriatric Services Resource Team (GSRT) was established in 2017 to introduce specialist interprofessional team-based assessment on an outpatient basis in the form of a comprehensive geriatric assessment (CGA) for patients presenting with falls, impaired mobility, functional decline, persistent pain or symptoms of dementia [14,15]. One of its purposes is to diagnose, review mechanisms of improving patient outcomes, and provide guidance on care planning and the education of caregivers for patients with dementia. The GSRT serves both urban and rural areas in southern Saskatchewan, covering a significant portion of the province. However, its impact on dementia diagnosis and management has yet to be evaluated. Given its large catchment area and the increasing need for the comprehensive management of dementia and MCI in Canada, we aimed to evaluate the GSRT’s impact on dementia care through the CGA use. Thus, the purpose of this study was to assess the CGA impact by the GSRT on the diagnosis and management of patients with dementia, compared to standard care provided in Primary Care (PC) settings.

## 2. Methods

### 2.1. Data Collection

Two clinical databases, PC (2010–2017) and GSRT (2018–2019), were subjected to secondary data analysis. Since the GSRT was established in 2017, we were not able to include a comparable PC time frame. Additionally, we made deliberate efforts to ensure that patients were not included in both the PC and GSRT cohorts. The PC practice evaluated in this study had nine family physicians, three nurses and one psychologist. It provides regular clinics and a walk-in service, representative of the average PC service in Regina. We believe this represents a typical Primary Care service provided by family doctors in Canada. The digital medical health record database (ACCURO) was searched with three key words, “dementia”, “cognitive impairment” or “memory loss”, and data from 90 PC patients’ charts were collected. GSRT referrals were made by family doctors or nurse practitioners for patients with complaints of cognitive impairment or those who had diagnosis of dementia but required further evaluation. Triage was done by a nurse coordinator, who ensured the reason for referral was adequate, and collected background information on medical history and baseline investigations to rule out reversible causes. The wait time for a CGA varied between 1 and 3 months. The CGA of patients was done with an initial home visit by a team member, after initial referral to the GSRT. This was followed by a clinic appointment with an inter-disciplinary team of a physician, a social worker, an occupational therapist (OT), a pharmacist, and a nurse, and archived in the digital medical records in the Regina Qu’Appelle Health Region, called Sunrise clinical manager (SCM), while some of the information was reviewed from the paper charts. The assessment included 1 to 2 visits by the inter-disciplinary team, followed by the provision of recommendations and signposting to resources in the community. Seventy-five consecutive clients with “dementia”, “cognitive impairment” or “memory loss” were identified and included in further analysis. The inclusion criteria comprised patients over the age of 65 years of age, having a diagnosis of dementia and/or referred for diagnosis and management of dementia. Patients were excluded if they lived in long-term care homes, were under the age of 65 years or had delirium. The same criteria were used for patients selected in PC.

This study was approved by the Regina Qu’Appelle Health Region Behavioral Research Ethics Board (REB-18-16).

### 2.2. Clinical Diagnosis, Interventions and Provided Service

Information on specialties involved in dementia diagnosis, forms of dementia, duration of complaints until dementia diagnosis, type of pharmacological and non-pharmacological interventions, and involvement of assisting occupations in support of health service were analyzed. The DSM-5 diagnoses dementia as a Major Neurocognitive Disorder (MND), defined by significant cognitive decline from a previous level in one or more domains (memory, executive function, language, etc.). This decline must interfere with independence in daily activities and not occur solely during delirium [16].

## 3. Data Analysis

Descriptive statistics characterized patients and services provided in the PC setting and by the GSRT. Student’s *t*-test was used to compare patients’ age between groups. Other variables (frequency (*n*, %)) were compared using Chi square tests of independence for contingency tables and post hoc pairwise comparisons for all group comparisons with cell counts > 5. All statistical tests were two-sided and conducted at the 5% significance level for primary tests using SPSS v28. Bonferroni corrections were applied for tests for all adjusted pairwise comparisons within each row.

## 4. Results

### 4.1. Demographics

Patient age was comparable between two services (PC: mean = 79.2 (SD = 8.14) years old; GSRT: mean = 76.8 (SD = 9.43) years old). Although no difference was observed for biological sex, 53 of the 90 (59%) PC patients and 50 of the 75 (67%) GSRT patients were female.

### 4.2. Dementia Diagnosis in Primary Care and by Geriatric Services Resource Team

Dementia was diagnosed by different specialists in the PC setting along with family physicians (χ^2^ = 112.326, df = 4, *p* < 0.001) (Table 1), suggesting family doctors preferred to refer to specialists to make a diagnosis of dementia; however, most of the diagnoses of dementia were made by the family physician under the GSRT (*p* < 0.001). We note that two patients were unaccounted for in each of the PC and GSRT group, where it is unclear who made the diagnosis.

### 4.3. Incidence of Different Forms of Dementia and Other Psychiatric Conditions

Several differences are found in the proportion of different diagnoses between groups (χ^2^ = 47.79, df = 7, *p* < 0.001). Half of the patients supported by the PC service received a dementia diagnosis compared to those served by the GSRT (*p* < 0.001) (Table 2), whereas patients referred to the GSRT predominantly suffered from Alzheimer’s Dementia (*p* < 0.05), Mild Cognitive Impairment (*p* < 0.01) and Vascular Dementia (*p* <0.05). In both groups, some patients were given multiple diagnoses. In the PC service, these were depression/anxiety and dementia in 7.8%, and memory impairment and MCI in 2.2%. In GSRT, 2.7% had a diagnosis of depression/anxiety with dementia.

### 4.4. Duration of Complaints Until Dementia Diagnosis

Analysis of the duration of complaints reveals overall group differences (χ^2^ = 24.59, df = 8, *p* = 0.02). Over 50% of the participants in PC were diagnosed as having dementia within a year of presentation as compared to just over 25% in GSRT (Table 3), with a *p*-value of <0.001. The overall trend showed that patients had a longer duration of symptoms prior to diagnosis in the GSRT group.

### 4.5. Pharmacological and Non-Pharmacological Interventions

Memory loss medications and antipsychotics were prescribed to significantly more patients with clinical care in the PC settings compared to the GSRT care (*p* < 0.001) (Table 4). Alternatively, lifestyle advice was more common for the GSRT than PC (*p* < 0.001). The prescription rate for antidepressants was comparable between GSRT and PC (*p* > 0.05).

### 4.6. Inter-Disciplinary Team Input in Support of Primary Care Services and Geriatric Services Resource Team

A significantly higher number of clients in the GSRT setting received support from OTs than the clients in PC (97.3% and 1.1%, respectively; *p* < 0.001) (Table 5). Social worker involvement was also statistically higher in GSRT (92%) than in PC (5.6%) (*p* < 0.001). Up to 90% of patients were consulted by pharmacists in the GSRT and PC service (*p* > 0.05).

SWADD involvement (System Wide Admission and Discharge Department), responsible for home care input and long-term care placement, was documented only for the GSRT and 17.3% of clients were assisted by them.

## 5. Discussion

This is one of the few studies comparing CGA care with the standard care for dementia in the province of Saskatchewan. It must be noted that while CGA plays a specific role in managing geriatric patients across Canada, care provided at the provincial level might vary due to factors such as demographics, geography, founding models and policies [6] impacting both the input and outcome of the CGA regarding the diagnosis and care provided.

One of the key findings was the diagnosis of MCI and the dementia subtype given to the patients in GSRT; for example, 84% of patients seen by the GSRT were diagnosed with MCI, Alzheimer’s Dementia, Vascular Dementia or Fronto-Temporal Dementia, as opposed to only 40% by PC. Similarly, over 50% of patients seen in PC received a diagnosis of Dementia, compared to only 9% of patients seen by the GSRT. This correlates with other published research where the CGA yielded higher diagnostic accuracy than a simple application of MoCA [17]. In an ideal world, an accurate diagnosis can have major clinical implications, allowing for the timely initiation of a tailored treatment approach such as acetylcholinesterase inhibitors, advice on lifestyle modifications and planning for future care and finances. It also enables patients to access other clinical and administrative resources based on theirs and their caregiver’s needs, such as the System Wide Admissions and Discharge Department (SWADD), to access a long-term care facility and other community resources. The current literature further corroborates this point.

Another key takeaway from this study was the difference in the management of cognitive and mental health impairments by the GSRT in contrast with PC. While fewer patients were prescribed memory loss medication and antipsychotics by the GSRT, more patients received lifestyle advice (e.g., diet, cardiovascular risk factors) including referral to the adult day programs. These lifestyle modifications, including regular exercise, a Mediterranean diet, smoking cessation, and avoiding the use of excess alcohol are known to reduce the risk of dementia in older people [18]. Moreover, a focus on appropriate lifestyle modifications before initiating pharmacotherapy can reduce the risk of polypharmacy. It also reduces the risk of cardiovascular disease, diabetes mellitus, cerebral-vascular accidents and hypercholesterolemia, which, as reported by Nordestgaard et al., are known risk factors for dementia that can be easily preventable with lifestyle measures [19].

As mentioned earlier in the discussion, more patients were given a diagnosis of MCI, Vascular Dementia, FTD and severe dementia. This is the likely reason why acetylcholinesterase inhibitors or glutamate receptor antagonists were not initiated in GSRT. We also report that the referrals to the GSRT for a CGA were made later in the course of the illness, by the family doctors, which likely resulted in delayed assessments and diagnoses of dementia. This delay could be the result of multiple factors including a lack of access to specialized teams prior to the establishment of the GSRT. There may also be a need for family doctors to receive further training in the diagnosis of different types and stages of dementia. Depending on access, an earlier referral can be made to specialist teams that are experienced in managing the complex health issues of the elderly. This will be particularly helpful in those situations where clarity on diagnosis and information on community services is not present. This study identifies the need for clear care pathways for patients with dementia, which, if followed, will allow for a standardized treatment for patients with dementia in the province of Saskatchewan.

The GSRT evaluated in this study utilizes the CGA principles that aims to detect unidentified and potentially reversible problems and develop a coordinated and integrated management plan for treatment and long-term follow-up care [20]. The healthcare of an older adult extends beyond the traditional medical management of illness. It requires an evaluation of multiple issues, including physical, cognitive, affective, social, financial, environmental, and spiritual components that influence an older adult’s health. Systematic evaluation of frail elderly persons may identify a variety of treatable health problems and lead to better health outcomes [21]. Our results showed an interprofessional CGA had a positive impact on dementia diagnosis, treatment interventions and future care planning.

## 6. Limitations

We would like to acknowledge a few limitations in our study design. The study was a retrospective analysis of the medical records. Thus, the findings were dependent on available records. It is possible that some aspects of care were inadequately recorded and, hence, not considered in this study. Data was accessible across several years in PC, whereas GSRT data was limited to two years due the fact that this service was new. Hence, no data was available from CGAs by the GSRT prior to the study period. We were aware that some of the patients seen in PC could have been referred for a CGA later in the course of their illness. One patient from the PC group was referred to the GSRT. As the comparison is about the level of service provision, the impact on diagnosis and further intervention would not have been affected. Despite the above-mentioned limitation, the findings remained unaffected, owing to the analysis of a comparable number of clients treated by each program.

Although there is evidence for comprehensive geriatric assessments in a variety of old age-related issues including frailty, falls, chronic disease and pain management, we only focused on the care of patients with cognitive impairment [22]. This means that further research on other comorbidities in dementia patients is warranted, especially given the underrepresentation of said patients in research. Further studies to evaluate the long-term impact of CGAs will help us find the impact of both care models on patient outcomes in the future.

## 7. Conclusions

CGAs provide an in-depth assessment and management for a variety of conditions in the aging population. This process can be utilized in patients with dementia or the dementia prodrome. The care and advice provided by a multidisciplinary team, with the focus of non-pharmacological as well as pharmacological interventions, ensures an optimal and holistic approach to the needs of elderly patients with dementia.

## Figures and Tables

**Table 1:** Contingency table of specialties involved in dementia diagnosis.

	GSRT	Primary Care	Total
Family Doctor	65 (90.4%) ***	42 (47.7%)	107 (66.9%)
Internal Medicine	0 (0.0%)	29 (33.0%)	29 (18.1%)
Neurology	0 (0.0%)	11 (12.5%)	11 (6.9%)
Psychiatry	1 (1.4%)	6 (6.8%)	7 (4.3%)
Other (RN/NP)	6 (8.2%)	0 (0.0%)	6 (3.8%)
Total	72 (45.0%)	88 (55.0%)	160 (100.0%)

Significant pairwise comparisons bolded, *p*, *** < 0.001. Tests of pairwise comparisons are adjusted using Bonferroni correction within each row.

**Table 2:** Contingency table of different forms of dementia.

	GSRT	Primary Care	Total
Mild Cognitive Impairment	21 (28.0%) **	10 (11.1%)	31 (18.9%)
Alzheimer’s Dementia	26 (35.2%) *	19 (21.1%)	45 (27.4%)
Fronto-Temporal Dementia	3 (4.0%)	1 (1.1%)	4 (2.4%)
Vascular Dementia	13 (17.6%) *	6 (6.7%)	19 (11.6%)
Severe Dementia, Mixed Type	2 (2.7%)	0 (0.0%)	2 (1.2%)
Dementia	7 (9.5%)	52 (57.8%) ***	59 (36.0%)
Memory Problems	0 (0.0%)	2 (2.2%)	2 (1.2%)
Other	2 (42.7%)	0 (0.0%)	2 (1.2%)
Total	74 (45.1%)	90 (54.9%)	164

Note: Majority of diagnoses in Primary Care service delivery were made by specialists in contrast to the physician for the Geriatric Services Resource Team. Significant pairwise comparisons bolded, *p* * < 0.05. ** < 0.01, *** < 0.001. Tests of pairwise comparisons are adjusted using Bonferroni correction within each row.

**Table 3:** Contingency table of duration of complaints until dementia diagnosis.

	GSRT	Primary Care	Total
Less than 12 months	19 (25.3%)	53 (58.9%) ***	72 (43.6%)
12–23 months	11 (14.7%) *	4 (4.4%)	15 (9.1%)
24–35 months	9 (12.0%)	7 (7.8%)	16 (9.7%)
36–47 months	8 (10.7%)	3 (3.3%)	11 (6.7%)
48–59 months	3 (4.0%)	2 (2.2%)	5 (3.0%)
60–71 months	5 (6.7%)	2 (2.2%)	7 (4.2%)
72–83 months	2 (2.7%)	1 (1.1%)	3 (1.8%)
More than 84 months	4 (5.3%)	1 (1.1%)	5 (3.0%)
Unknown	14 (18.7%)	17 (18.9%)	31 (18.8%)
Total	75 (45.5%)	90 (54.5%)	165 (100.0%)

Significant pairwise comparisons bolded, *p* * < 0.05. *** < 0.001. Tests of pairwise comparisons are adjusted using Bonferroni correction within each row.

**Table 4:** Contingency table of pharmacological and non-pharmacological interventions.

		GSRT (*n* = 75)	Primary Care (*n* = 90)	χ^2^	*p*-Value
Acetylcholinesterase inhibitors and Memantine	Yes No	53 (70.7%) 22 (29.3%)	77 (85.6%) 13 (14.4%)	5.43 df = 1	0.020
Antipsychotics	Yes No	1 (1.3%) 74 (98.7%)	16 (17.8%) 74 (82.2%)	11.971 df = 1	<0.001
Antidepressants	Yes No	10 (13.3%) 65 (86.7%)	19 (21.1%) 71 (78.9%)	1.708 df = 1	0.191
Lifestyle advice	Yes No	23 (30.7) 52 (69.3%)	2 (2.2%) 88 (97.8%)	25.746 df = 1	<0.001

**Table 5:** Contingency table of inter-disciplinary team input.

		GSRT (*n* = 75)	Primary Care (*n* = 90)	χ^2^	*p*-Value
Occupational Therapist	Yes No	73 (97.3%) 2 (2.7%)	1 (1.1%) 89 (8.9%)	153.132 df = 1	<0.001
Social Worker	Yes No	69 (92.0%) 6 (8.0%)	5 (5.6%) 85 (94.4%)	123.592 df = 1	<0.001
Pharmacist	Yes No	67 (89.3%) 8 (10.7%)	75 (83.3%) 15 (16.7%)	1.228 df = 1	0.268
SWADD	Yes No	13 (17.3%) 62 (82.7%)	Missing	N/A	N/A

N/A: not applicable.

## Data Availability

Restrictions apply to the availability of these data. Data were obtained from Saskatchewan Health Authority (SHA) and are available (shazia.durrani@saskhealthauthority.ca) with the permission of SHA.
